# Unveiling the antibacterial action of ambroxol against *Staphylococcus aureus* bacteria: in vitro, in vivo, and in silico investigation

**DOI:** 10.1186/s12866-024-03666-x

**Published:** 2024-11-29

**Authors:** Ahmed A. Abdelaziz, Amal M. Abo-Kamar, Alaa E. Ashour, Moataz A. Shaldam, Engy Elekhnawy

**Affiliations:** 1 Pharmaceutical Microbiology Department, Faculty of Pharmacy, Tanta University, Tanta, 31527 Egypt; 2 Department of Pharmaceutical Chemistry, Faculty of Pharmacy, Kafrelsheikh University, Kafr El-Sheikh, 33516 Egypt

**Keywords:** Biofilm, Resistance, Morphology, Gene expression, Molecular docking

## Abstract

It is critical to find novel therapeutic approaches owing to the dissemination of multidrug resistance (MDR) in pathogenic bacteria, particularly *Staphylococcus aureus*. FDA-drug repurposing is an important therapeutic tactic to fight MDR bacteria. Here, we inspected the antibacterial activity of ambroxol against clinical MDR *S. aureus* isolates. Using the broth microdilution method, ambroxol revealed minimum inhibitory concentrations (MICs) of 0.75 to 1.5 mg/mL. Also, it revealed antibiofilm action on 42.17% of the isolates by crystal violet assay. A scanning electron microscope was employed to study the antibiofilm action of ambroxol. It revealed that the association between the cells was interrupted by ambroxol, and the biofilm construction was devastated. Moreover, qRT-PCR was utilized to elucidate the consequence of ambroxol on the gene expression of efflux and biofilm. Remarkably, ambroxol has downregulated the expression of *cna*, *fnb* A, *ica*, *nor* A, *nor* B genes. Ambroxol’s in vivo antibacterial action was investigated using *S. aureus* infected burn infection. Interestingly, ambroxol has improved the histological features of the skin tissues, significantly diminished the bacterial burden, and increased the wound healing percentage. Also, it revealed a significant reduction in the immunohistochemical staining of tumor necrosis factor-alpha. Finally, the in silico investigations were performed to elucidate the potential of ambroxol on five possible targets of *S. aureus.* Ambroxol showed good affinities on the five investigated targets in *S. aureus*, with CrtM being the highest, proposing its probable role in the mechanisms for ambroxol’s action on *S. aureus.*

**Supplementary Information:**

The online version contains supplementary material available at 10.1186/s12866-024-03666-x.

## Introduction

One risk that threatens human health is multidrug-resistant (MDR) bacteria. In recent years, bacteria have resisted many approved antibiotics on the market that are used to treat severe infections caused by pathogenic bacteria [1]. One of the virulence factors that helps in bacterial resistance is biofilm formation. Bacterial biofilms help bacteria evade the immune system^’^s response and decrease their sensitivity to antibiotic treatment. So, it is necessary to find drugs with an inhibitory effect on biofilm formation [2].

Antimicrobial resistance increases the hospitalization period, healthcare expenses, morbidity, and mortality. Infection caused by *Staphylococcus aureus* is a chief international healthcare problem because of its fast rate of developing resistance to common antibiotics [3]. Thus, new approaches are required to fight against these pathogens like repurposing the FDA-approved drugs to treat such bacterial infections. Repositioning the FDA-approved drugs is a hopeful tactic as new drug discovery needs a lot of time and cost, with a high failure rate [4].

*S. aureus* acts as a commensal of human microbiota but can become an opportunistic pathogen. It can cause different ailments like bacteremia and infections in the endocardium, skin and soft tissue, and osteoarticular system [5]. The multiple virulence factors of *S. aureus* and overconsumption of antibiotics have led to great difficulty in treating such infections [6].

*S. aureus* has various virulence factors, such as teichoic acids, clumping factors, fibrinogen-binding proteins, Staphylococcal superantigens (TSST-1 or toxic shock syndrome toxin 1), PVL (Panton-Valentine leukocidin), Protein A, alpha-hemolysin, and biofilm [7]. In the current study, we aimed to find an alternative treatment for MDR *S. aureus* by repurposing ambroxol for *S. aureus* infections.

## Materials and methods

### Bacteria

Bacterial samples were taken from patients of Mansoura University Hospital, Mansoura, Egypt, from different sources (sputum, blood, wound, throat, nose, ear, cornea, and urine). The isolated bacterial colonies were identified by Gram-staining and biochemical identification [8]. S *taphylococcus aureus* ATTC 25,913 was employed as a standard strain, and 83 *S. aureus* isolates were tested. The media and chemicals were from Oxoid, UK, and Merck, USA.

### Antibiotic sensitivity test

The antibiotic sensitivity of the *S. aureus* isolates was applied using Kirby-Bauer disk diffusion. Mueller-Hinton agar (MHA) plates are used in this method [9–11]. The following antimicrobials were used, oxacillin (OX; 1 µg), erythromycin (E; 15 µg), gentamicin (GN; 10 µg), linezolid (LZD; 30 µg), clindamycin (DA; 2 µg), tetracycline (TE; 30 µg), trimethoprim-sulfamethoxazole (COT; 1.25/23.75 µg), minocycline (MI; 30 µg), gatifloxacin (GAT; 5 µg), chloramphenicol (C; 30 µg), azithromycin (AZM; 15 µg), and ciprofloxacin (CIP; 5 µg).

### Antibacterial action of ambroxol

It was revealed by agar well diffusion in MHA plates [12]. The bacterial suspension (0.5 McFarland) was dispersed on the surface of the MHA plates. Three wells were performed using a 5 mm diameter cork borer. The first well received ambroxol hydrochloride in water (3 mg/mL), the second received linezolid (positive control), and the third well received sterile water (negative control). The appearance of inhibition zones revealed the antibacterial activity of ambroxol after incubating the plates at 37 °C for 24 h [13–15].

### Determination of the minimum inhibitory concentration (MIC) of ambroxol

Broth microdilution assay in MH broth was employed to estimate the MIC values of ambroxol against the MDR *S. aureus* isolates, as previously reported [13]. MIC was the lowest concentration of ambroxol that visually displayed no growth was detected after overnight incubation at 37 °C [16].

### Biofilm inhibition

The biofilm formation assay was applied following the method of Stepanovic et al. [17] (supplementary file) [13, 18–20].

### Scanning electron microscope (SEM)

The antibiofilm action of ambroxol on *S. aureus* bacteria was visualized under SEM as previously explained SEM (JEOL, Japan) [21].

### Gene expression measurement using qRT‑PCR

The consequence of ambroxol was studied on the expression levels of the biofilm and efflux genes (*cna*, *fnb* A, *ica*, *nor* A, *nor* B) by qRT-PCR (supplementary file). Primers are listed in Table S1 [22, 23].

### Burn infection model

Twenty-four white albino male rats weighing 120–150 g with an age ranging from 6 to 8 weeks were employed in this study [24, 25]. They were purchased from the faculty of veterinary medicine at Cairo University. The protocol of this experiment was approved by the research ethical committee of the faculty of pharmacy, Tanta University, Egypt (TP/RE/2/24 p-04). The burn was created using a 10 mm diameter cylindrical metal rod. The metal rod was heated and pressed for 10 s on the dorsal mouse skin surface after hair shaving under anesthesia. Rats were randomly divided into four groups; group I: normal control, group II (positive control): infected burn wound and administered saline (0.9%), group III: infected burn wound and administered silver sulphadiazine (1%) as a standard drug, topically twice daily, group IV: infected burn wound and administered ambroxol (10 mg/mL) topically twice daily [26].

Bacterial infection of the burn wounds was induced by inoculating 1.5 × 10^7^ colony-forming units (CFU) of bacteria into the wound. Using a sterile cotton swab, one milliliter of the bacterial suspension was inoculated evenly by directly rubbing it onto the fresh burn [27].

### Histopathological and immunological analysis

For histopathological analysis, tissue samples were excised after the rats were euthanized using isoflurane at a concentration of 5% delivered via a nose cone and inhaled. Then, samples were put in buffered formalin (10%), dehydrated with alcohol, and inserted into paraffin wax blocks [28, 29]. Hematoxylin and eosin (H&E) were used to stain thin sections of tissue samples (5 μm) for assessment of pathological alterations using a light microscope (MEIJI, USA).

Histopathological scoring of the skin tissues was blindly scored. The scale is: (1) the presence of polymorphonuclear neutrophils refers to inflammatory response; (2) the presence of fibroblasts, myofibroblasts, and neovascularization refers to granular tissue; (3) the density of collagen fibers refers to fibrosis. A score was made for all parameters: 0 = absent, 1 = mild presence, 2 = moderate presence, and 3 = strong presence [30]. Five random fields from each slide were analyzed.

The immunohistochemical analysis was accomplished using tumor necrosis factor-alpha (TNF-α) monoclonal antibody (Abcam, USA). For measuring the area percentage of the TNF-α immunohistochemical staining, the resulting images were analyzed by ImageJ software. Five random fields sized 200 × 200 μm from each slide were analyzed [31].

### Molecular docking

The crystal structures of target proteins of *S. aureus* from the protein data bank (PDB) were subjected to docking investigations by AutoDock Vina [32]. Ligand structures were drawn into MarvinSketch (version 23.17, by ChemAxon, USA), accessed on 5 Jan 2024, and the most energetically favored conformer was exported as a (*.pdb) file format. Molecules of water were removed, adding hydrogen assigned gastier charges were performed using AutoDock tools. The centers and sizes of the grid boxes used to define the active site for each receptor are exposed in Table 1. The 3D visualization and 2D schematic demonstration were produced by BIOVIA Discovery Studio Visualizer [33].

**Table 1:** Target proteins in *S. Aureus*, their PDB code and grid box information

Protein	Amnio-tRNA synthetase	FtsA	ClfA	NorA	Dehydrosqualene synthase (CrtM)
PDB (Ref)	1NYQ [34]	3WQT [35]	1N67 [36]	7LO7 [37]	4F6V [38]
Grid coordinates (x, y, z)	5.6, 53.3, 56.9	-4.7, 7.0, 19.1	28.5, 48.4, 81.5	134.0, 135.1, 157.2	25.7, -12.6, 8.5
Grid Size (x, y, z)	17.7, 23.6, 16.0	20.5, 23.8, 17.0	25.9, 27.4, 26.0	19.1, 31.1, 24.6	17.3, 27.5, 17.4

### Statistics

All tests were performed three times and showed mean ± standard deviation (SD). ANOVA was utilized to reveal the significance of differences among the experimental groups by GraphPad software (USA).

## Results

### Bacterial isolates and antibiotic sensitivity test

**Fig. 1:**
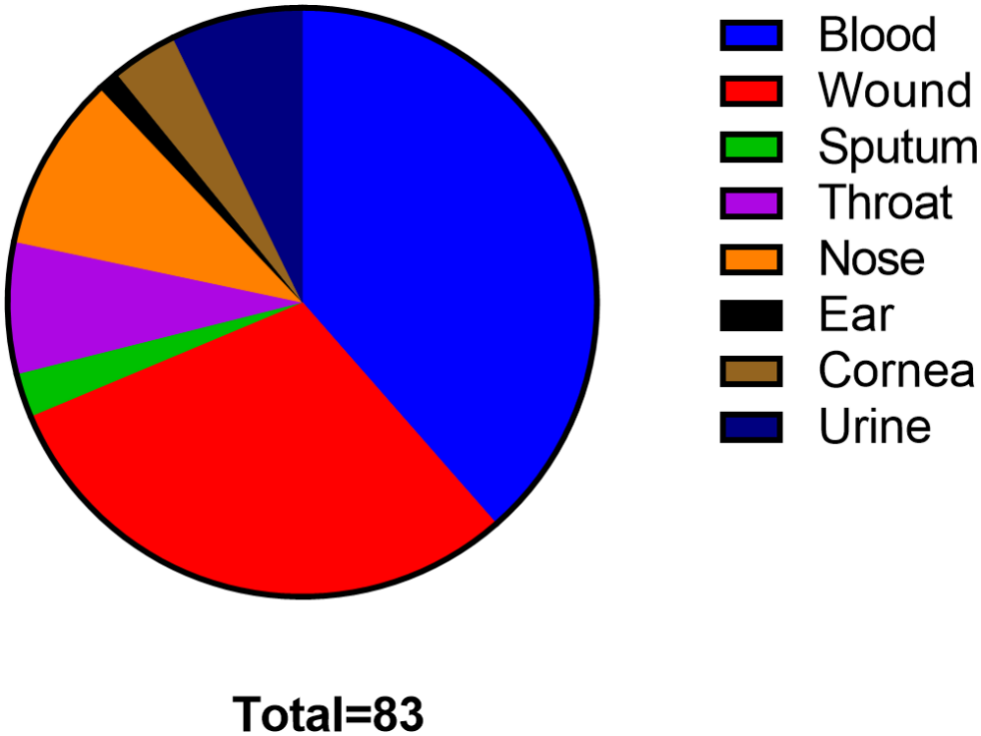
A pie chart representing the clinical specimens from which *S. aureus* isolates were isolated

**Fig. 2:**
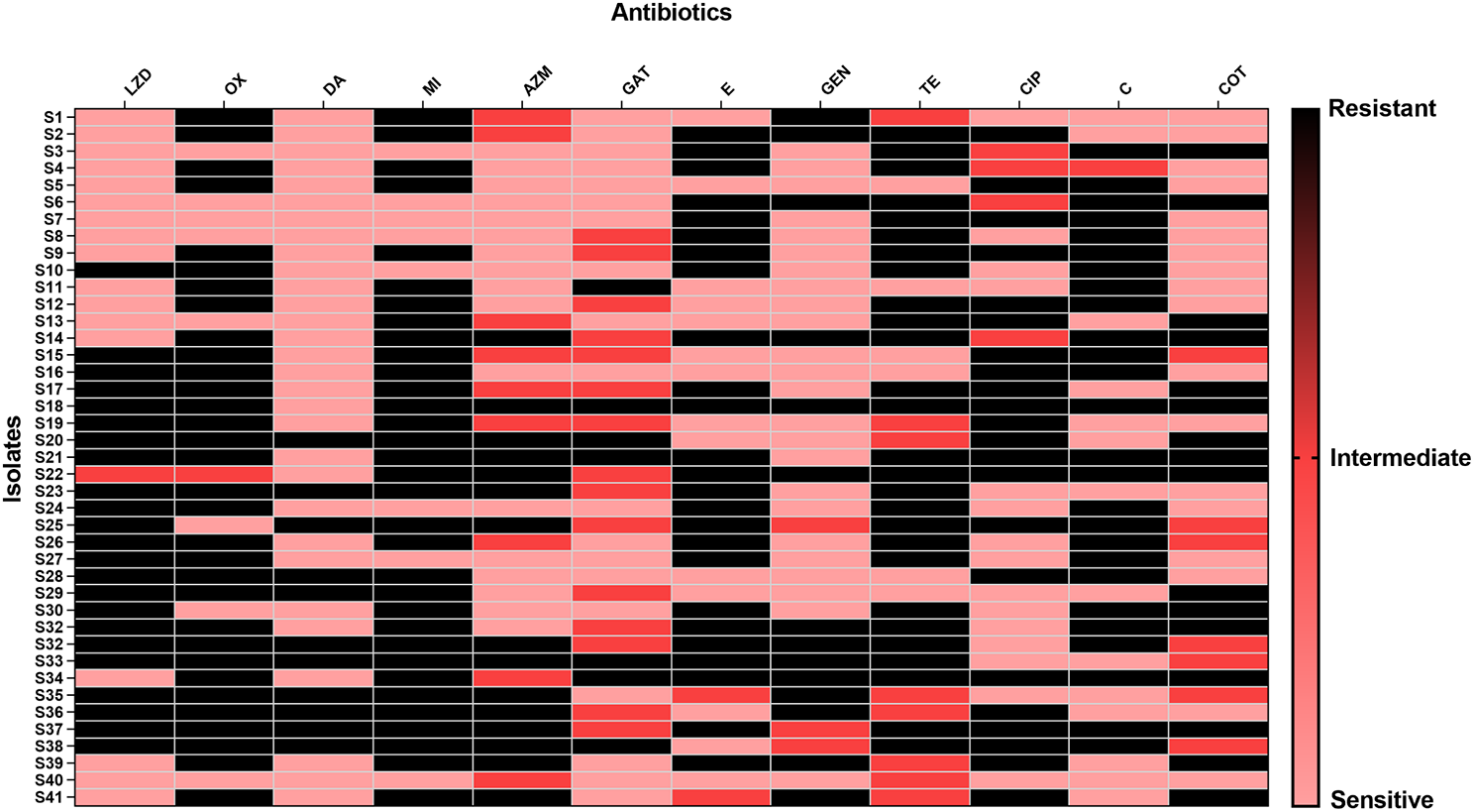
Heat map representing the antibiotic susceptibility of the isolates S1 to S41. Oxacillin (OX), azithromycin (AZM), erythromycin (E), chloramphenicol (C), gentamicin (GN), trimethoprim-sulfamethoxazole (COT), linezolid (LZD), clindamycin (DA), tetracycline (TE), minocycline (MI), gatifloxacin (GAT), and ciprofloxacin (CIP)

**Fig. 3:**
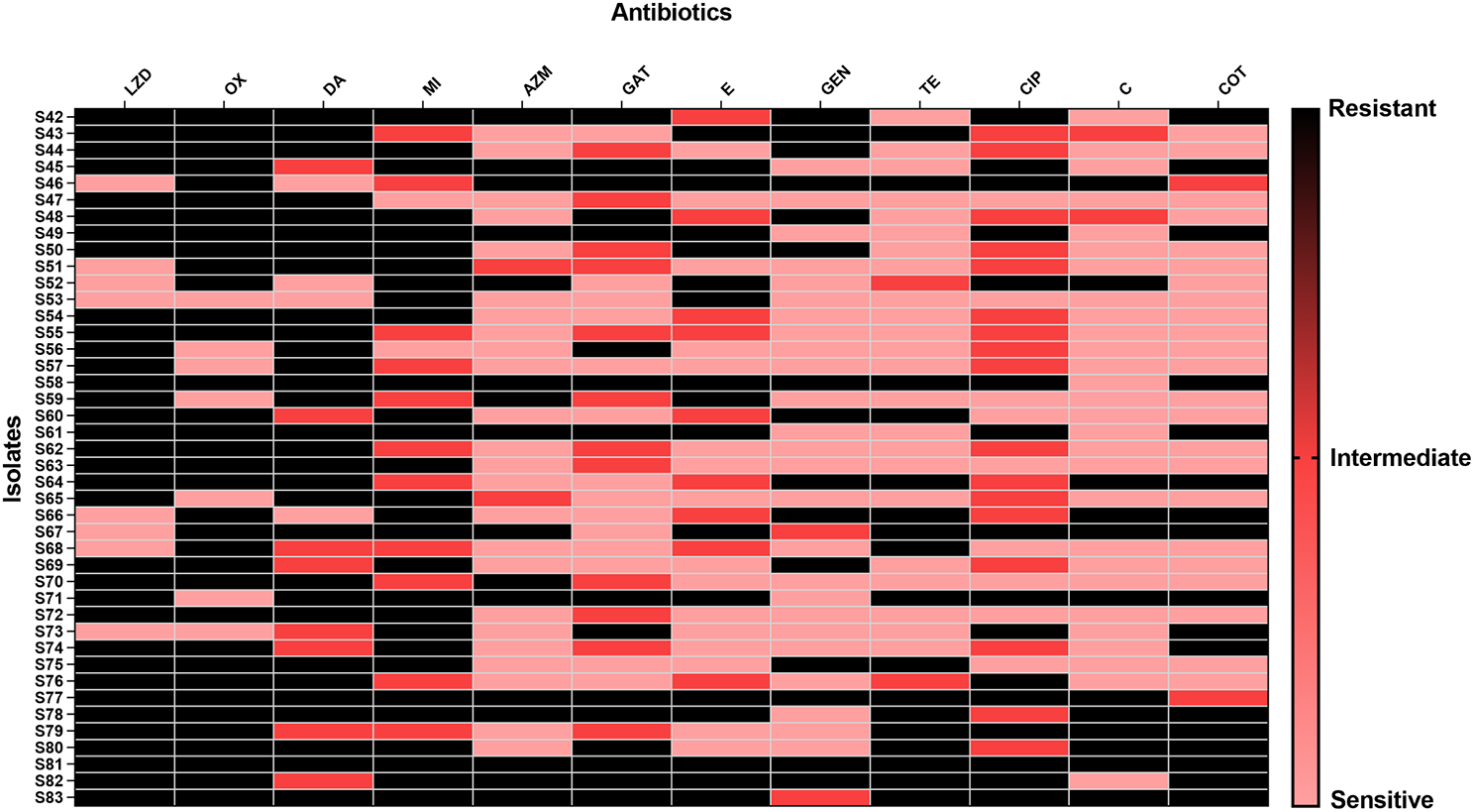
Heat map representing the antibiotic susceptibility of the isolates S42 to S83. Oxacillin (OX), azithromycin (AZM), erythromycin (E), chloramphenicol (C), gentamicin (GN), trimethoprim-sulfamethoxazole (COT), linezolid (LZD), clindamycin (DA), tetracycline (TE), minocycline (MI), gatifloxacin (GAT), and ciprofloxacin (CIP)

A total of 83 *S. aureus* bacteria were isolated from different specimens, including blood (32), wound (25), sputum (2), throat (6), nose (8), ear (1), cornea (3), and urine (6) as shown in Fig. 1. The resistance rates involved in the study were the following: oxacillin (82%), azithromycin (49%), erythromycin (53%), chloramphenicol (36%), gentamicin (45%), trimethoprim-sulfamethoxazole (31%), linezolid (54%), clindamycin (54%), tetracycline (52%), minocycline (43%), gatifloxacin (36%) and ciprofloxacin (39%) (Figs. 2 and 3).

### MICs of ambroxol and biofilm inhibition assay

The MICs of ambroxol ranged from 0.75 to 1.5 mg/mL, and ambroxol’s consequence on inhibiting biofilm formation was revealed. As shown in Table S1 and Figure S1, ambroxol has inhibited biofilm formation in 35 isolates (42.17%).

### SEM analysis

**Fig. 4:**
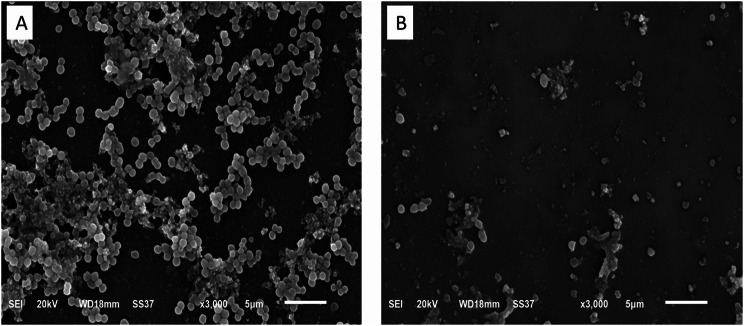
Impact of ambroxol on the biofilm formation using SEM

SEM was used to confirm the capacity of ambroxol to hinder biofilm formation. After using ambroxol, the number of bacterial cells decreased in the MDR biofilm-forming isolates. Ambroxol treatment disrupted the linking between the cells and destroyed the biofilm architecture compared to the untreated isolates (Fig. 4).

### qRT‑PCR

**Fig. 5:**
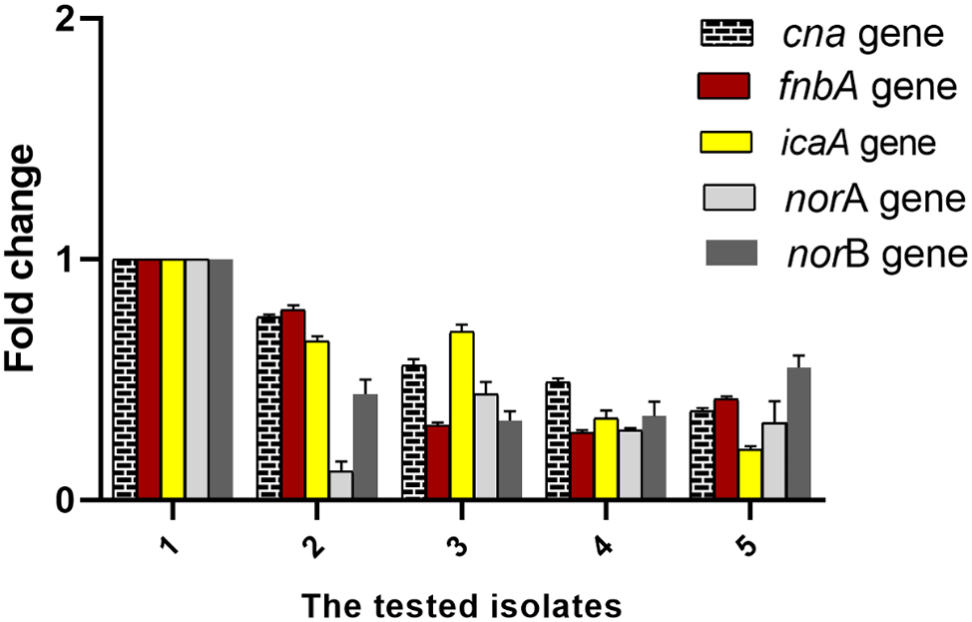
Fold change of the *cna*, *fnb* A, *ica* A, *nor* A, and *nor* B genes in the tested isolates after treatment with ambroxol

It was accomplished to identify the consequence of ambroxol on the efflux and the biofilm gene expression. Ambroxol significantly downregulated the efflux and biofilm genes in the tested isolates (Fig. 5, Figure S2).

### Healing of the wound

**Fig. 6:**
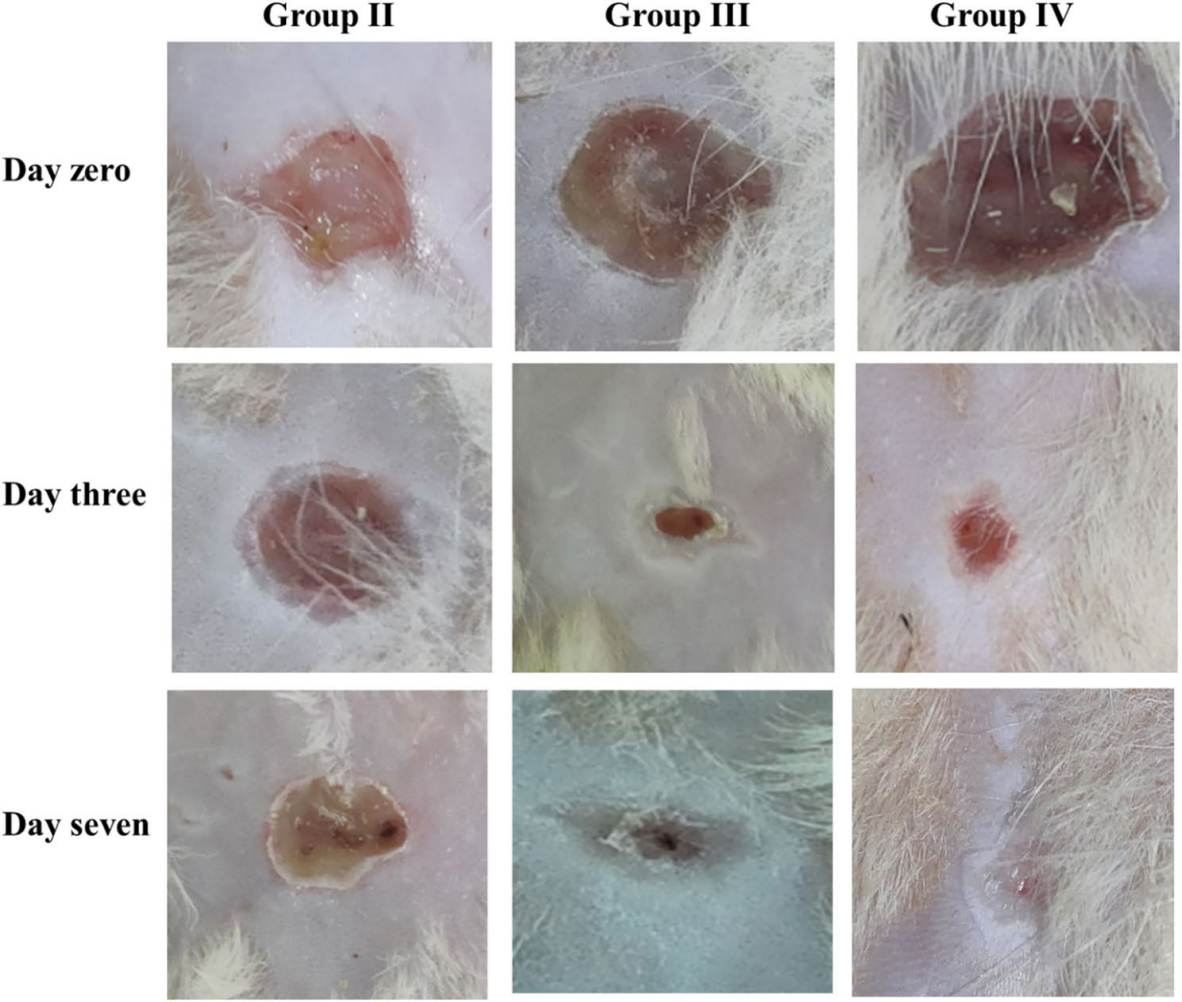
Macroscopic characters of the wounds of the different groups on days zero, three, and seven

The consequence of ambroxol on the MDR *S. aureus* infection was explored by induction of infection in a burn of rats by recording the burn closure in the groups. The percentage of the closure was considerably (*p* < 0.05) greater in the ambroxol-treated rats compared to untreated rats on different days of the experiment, which was comparable to the standard drug-treated group (Fig. 6).

**Fig. 7:**
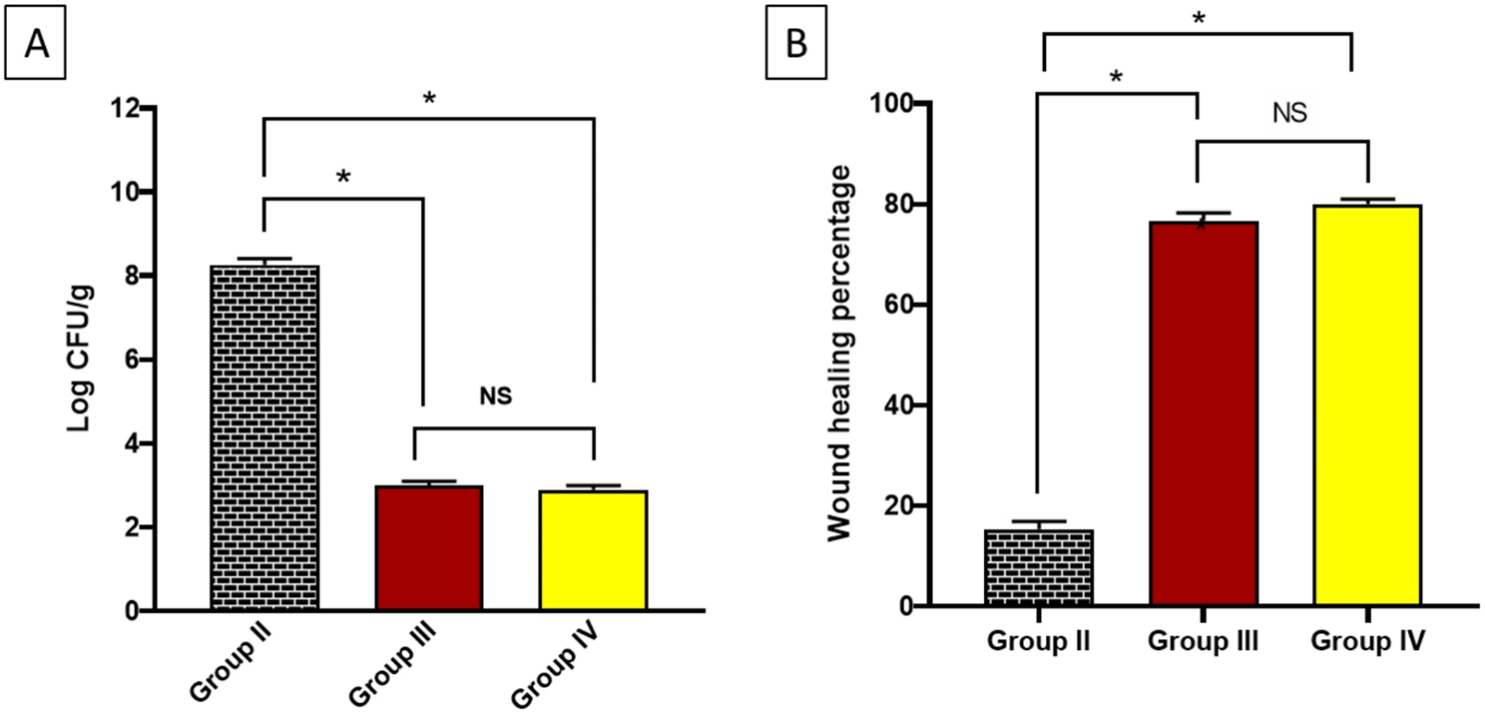
**A**) Bacterial burden in the experimental groups. **B**) Wound healing percentage of the experimental groups. The (*) denotes a noteworthy difference (*p* < 0.05), while NS symbolizes a non-significant (*p* > 0.05) difference

The efficiency of ambroxol was also investigated by investigating the bacterial burden in rat-infected skin burns. The treated wounds disclosed a considerably (*p* < 0.05) lower bacterial burden than the untreated wounds. Also, the wound healing percentage was calculated, and ambroxol improved wound healing significantly (Fig. 7).

### Histopathological and immunohistochemical analysis

**Fig. 8:**
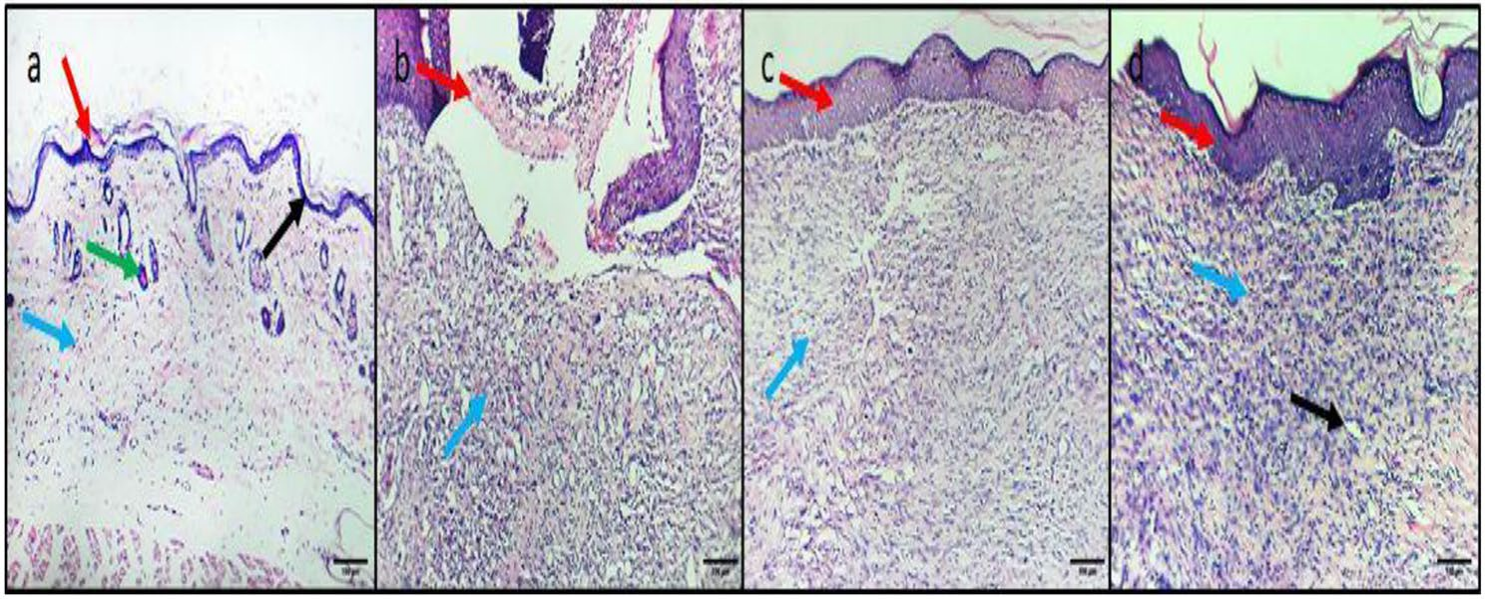
Photomicrographs of H&E-stained skin sections of **a**) Normal control group, showing normal histological skin arrangement with the superficial epidermis (red arrow) and deep dermis (blue arrow) with well-defined folding dermo-epidermal junctions (black arrow). The dermis is fibrous with no inflammation, and hair follicles (green arrow) with a central keratin core can be seen. **b**) Positive control group, showing an ulcer covered by a scab (red arrow) filled with acute and chronic inflammatory cellular infiltrate, granulation tissue with numerous small blood vessels, and fibrosis (blue arrow). **c**) Standard drug-treated group, showing complete re-epithelialization (red arrow), and the dermis has a few mononuclear cellular infiltrations, no granulation tissue, and no skin appendages (blue arrow). **d**) Ambroxol-treated group, showing complete re-epithelialization (red arrow), dermis with intense inflammatory cell infiltration (blue arrow), and few blood vessels (black arrow) with no skin appendages. 100× magnification

**Fig. 9:**
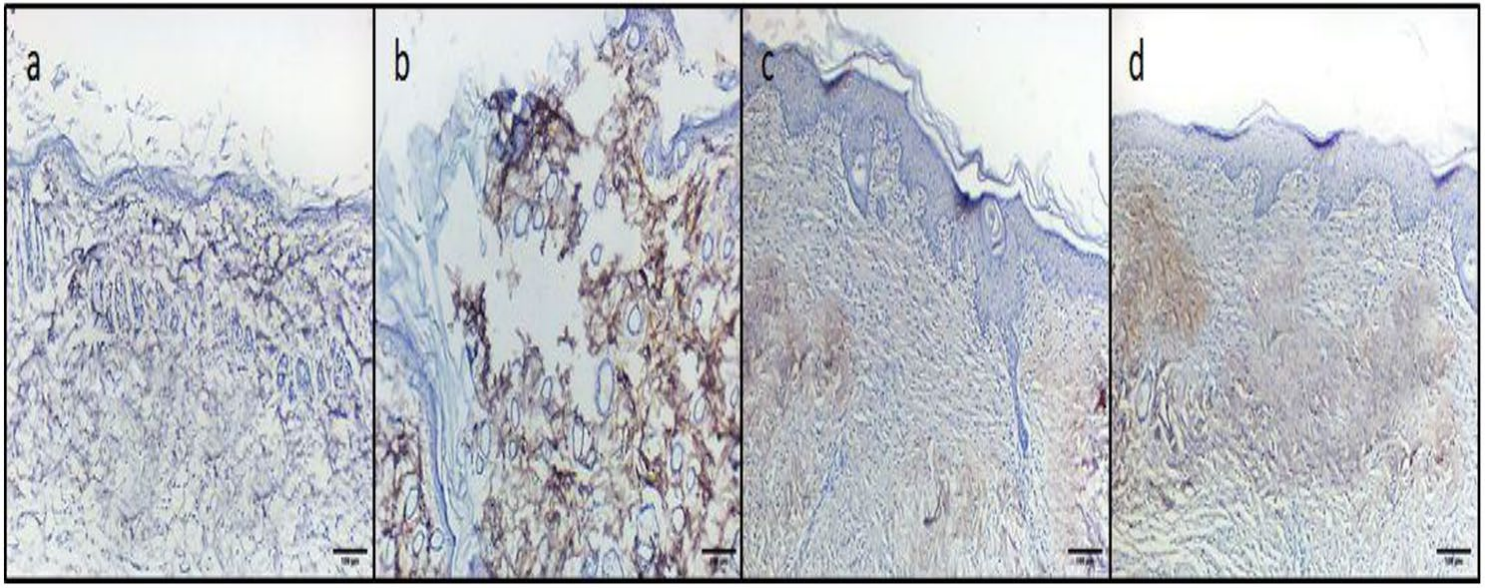
Photomicrographs of TNF-α immunohistochemical stained skin sections of **a**) Normal control group, showing minimal immunohistochemical reactivity for TNF-α. **b**) Positive control group, showing severe TNF-α immune reactivity. **c**) Standard drug-treated group, displaying mild immunohistochemical TNF-α reaction. **d**) Ambroxol-treated group, displaying a moderate TNF-α immune reactivity. 100× magnification

Ambroxol was found to significantly improve the histological architecture of the skin, as shown in Fig. 8. Also, the immunohistochemical studies of the TNF-α exposed a noteworthy reduction in the inflammatory marker (TNF-α), as shown in Fig. 9.

**Fig. 10:**
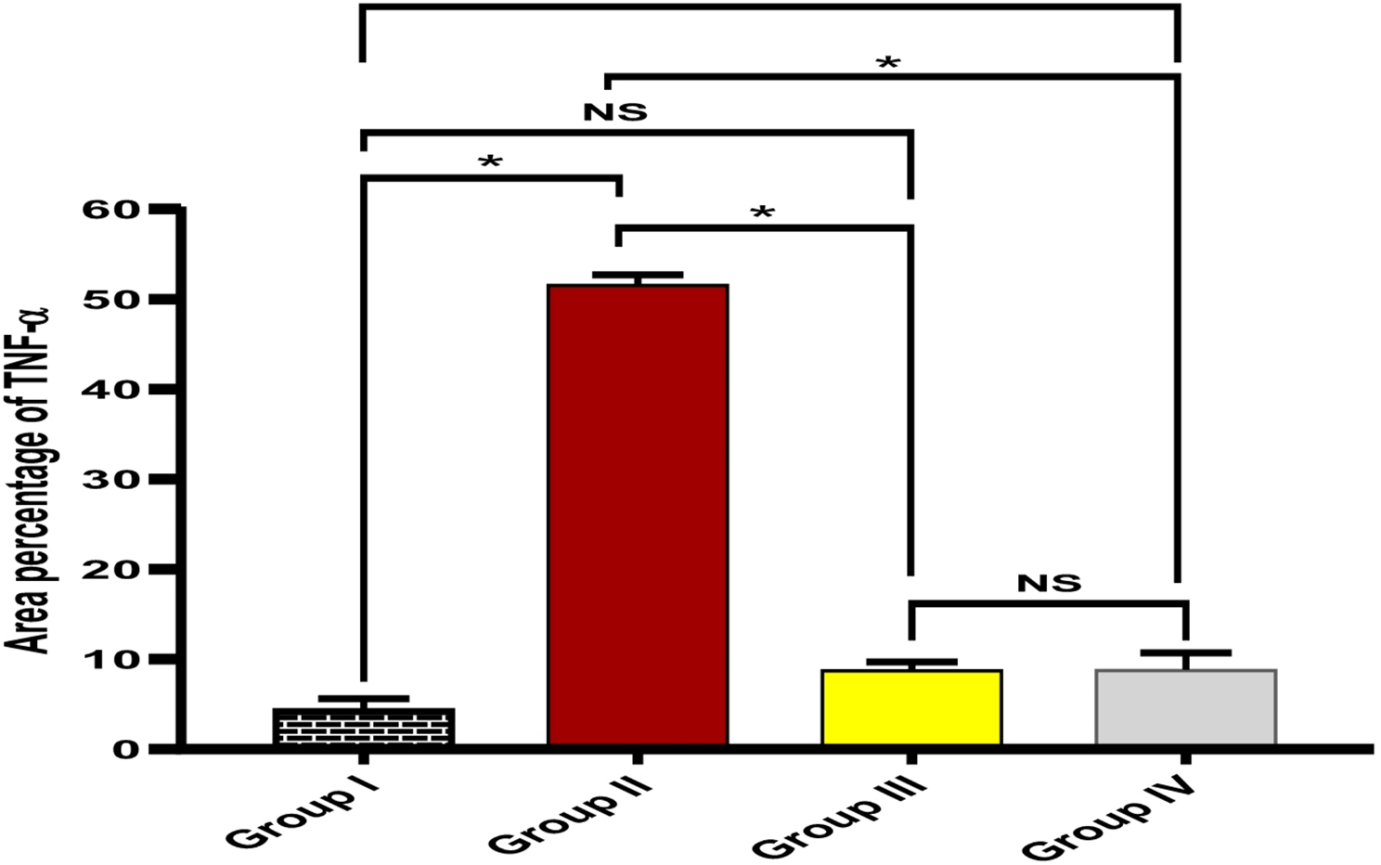
Bar graph displaying the percentage of the TNF-α immunostaining in the experimental groups. The (*) denotes a significant difference (*p* < 0.05), while NS symbolizes a non-significant (*p* > 0.05) difference

As shown in Fig. 10, ambroxol has decreased the TNF-α immunostaining surface area percentage. In addition, ambroxol has improved the polymorphonuclear neutrophils, granulation, and fibrosis of the skin sections (Table S3).

### Docking

The binding affinity and binding mechanisms for ambroxol on five proteins associated with.

*S. aureus* was inspected using molecular docking. The tested ligand displayed variable binding affinities to the target proteins under investigation, as designated by the docking score (Table 2).

**Table 2:** Docking affinities (Kcal/mol) for studied compounds into proteins of *S. aureus*

Protein	Amnio-tRNA synthetase	FtsA	ClfA	NorA	Dehydrosqualene synthase (CrtM)
**Docking affinities**	-6.5	-6.9	-7.3	-6.7	-7.5

**Fig. 11:**
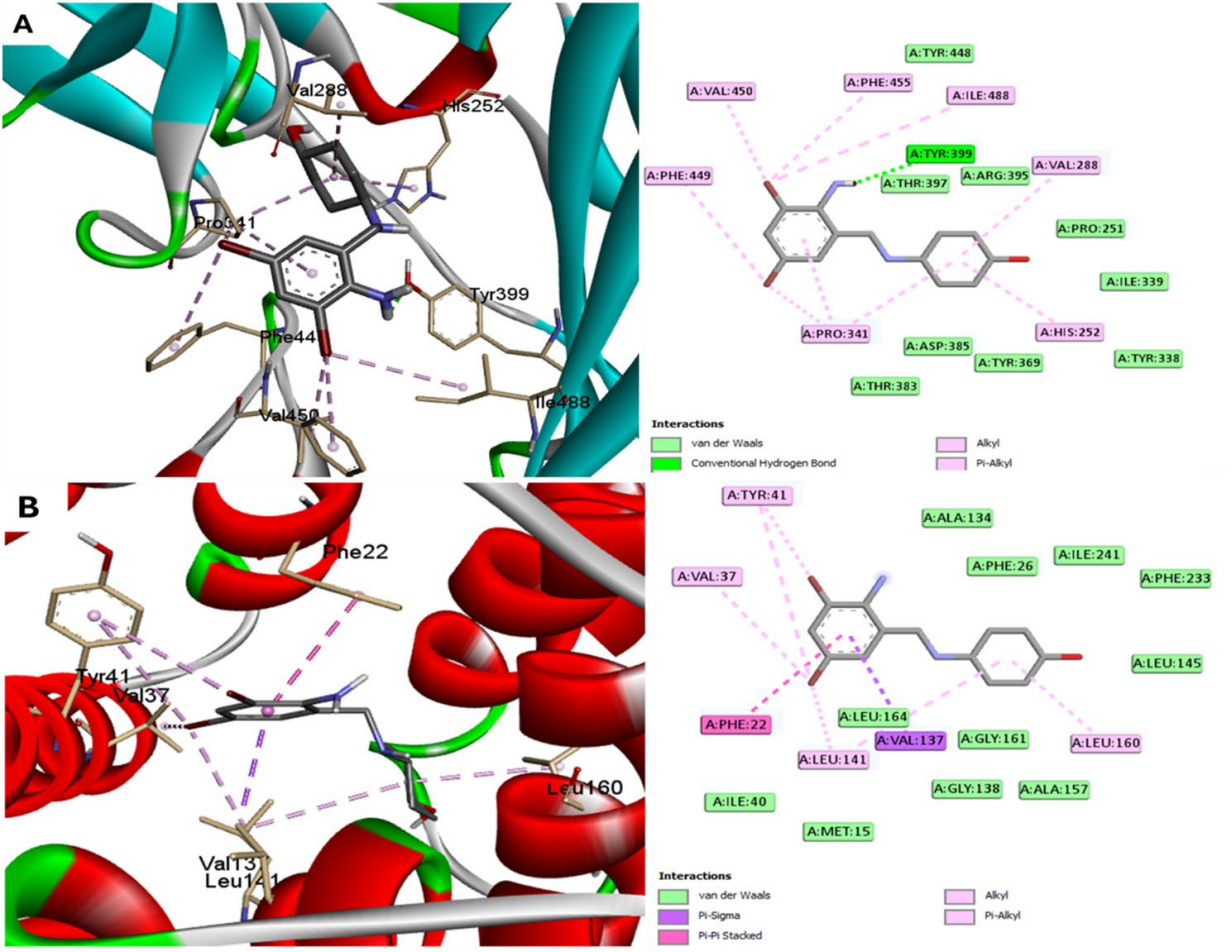
Docking of ambroxol into proteins of *S. aureus* (**A**) ClfA (code: 1N67) and (**B**) CrtM (code: 4F6V) enzymes

According to the docking affinities, ambroxol showed higher affinity to ClfA and CrtM enzymes. Different forces were involved, including H-bonding, hydrophobic interaction, Pi-sigma and Pi-Pi interactions (Fig. 11). An effective Pi-sigma with Val137 and Pi-Pi interactions with Phe22 were in charge of the high binding affinity of ambroxol with CtrM enzyme.

**Fig. 12:**
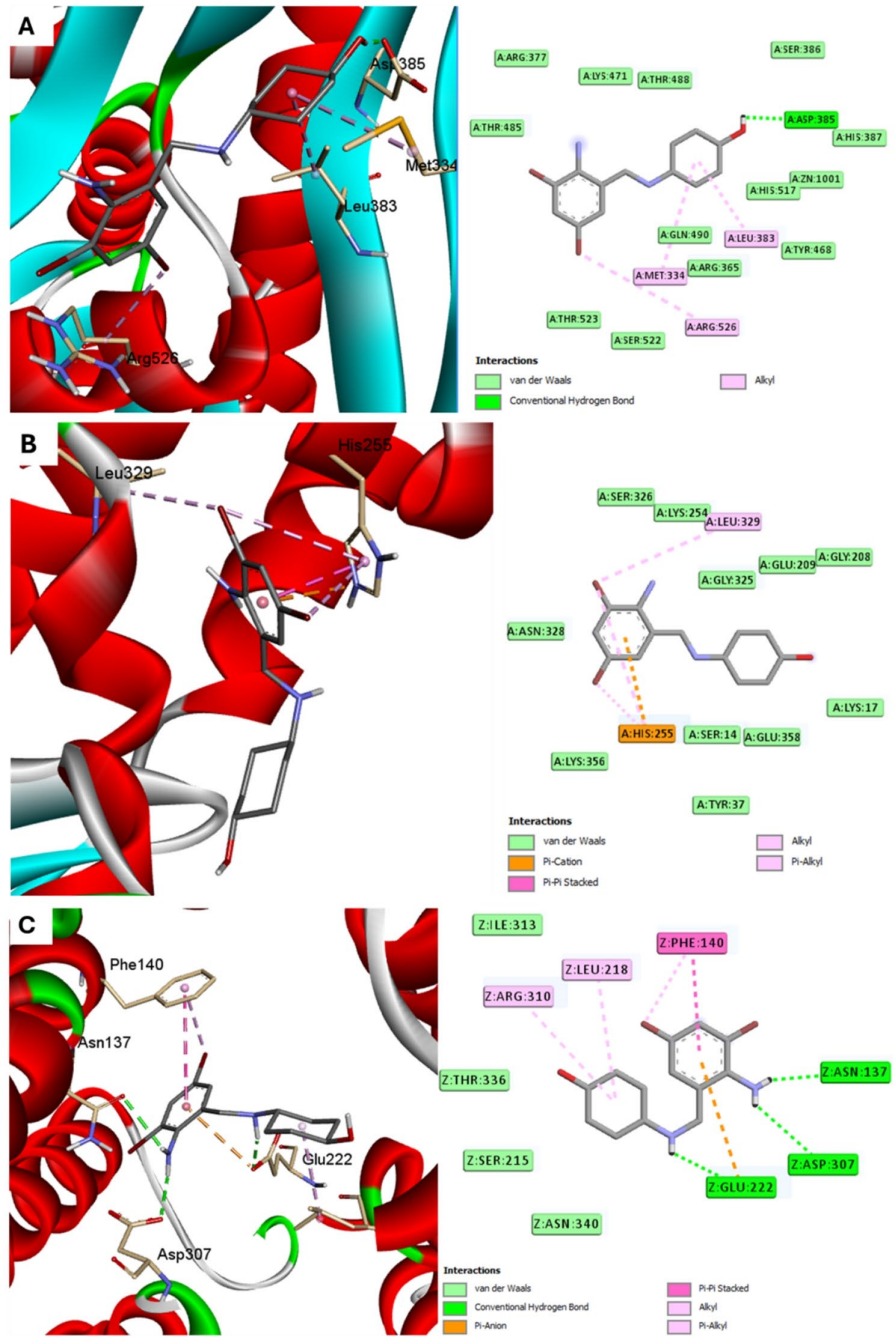
Docking of ambroxol into proteins of *S. aureus* (**A**) Amnio-tRNA synthetase (code: 1NYQ), (**B**) FtsA (code: 3WQT), and (**C**) NorA (code: 7LO7) enzymes

Regarding the other target, ambroxol showed nearly similar affinity with the following order FtsA > NorA > Amnio-tRNA synthetase. Different binding forces exert a variable degree of effect on affinity in each enzyme, which was reflected in the binding scores (Fig. 12).

## Discussion

The bacterial resistance to antibiotics is a major healthcare problem. This problem is increased because of the poor availability of novel antibiotics [39–41]. The resistant bacteria cause severe infections that lead to an increase in the length of hospitalization, healthcare costs, morbidity, and mortality. Thus, new orientations to solve this problem are required [42, 43]. Repurposing FDA-approved drugs to treat the infections is an alternative way to avoid time consumption that needs to arrive at new antimicrobials [44, 45].

Previous reports have investigated the capacity of different FDA-approved drugs like ambroxol for the management of various diseases, like sub-acute and chronic ischaemic stroke [46], neurodegeneration [47], and Gaucher disease [48]. Regarding the antimicrobial potential of ambroxol, a previous study revealed its effect on severe acute respiratory syndrome coronavirus 2 (SARS-CoV-2) [49].

*S. aureus* is one of the ESKAPE pathogens that include *S. aureus*, *Klebsiella pneumoniae*, *Enterococcus faecium*, *Pseudomonas aeruginosa*, Enterobacter spp., and *Acinetobacter baumannii*. Such pathogens are the main reason for healthcare-associated infections all over the world, and they are acquiring various antibiotic resistance mechanisms [50]. QS-regulated virulence factors are responsible for the capability of bacteria to trigger infections [51, 52]. So, various therapeutic strategies target these factors.

Antibiotic efflux by membrane transporters and biofilm formation are important for antibiotic resistance [53, 54]. *S. aureus* can form biofilms triggering severe infections due to antibiotic resistance that may reach 1000 times higher than in planktonic cells [55, 56].

In our study, ambroxol showed an ability to inhibit MDR isolates with a MIC value (0.75–1.5 mg/mL). We found that ambroxol had inhibitory activity against biofilm formation by crystal violet assay.

Moreover, SEM images confirmed that ambroxol disrupted MDR *S. aureus* biofilms, as treatment with ambroxol has decreased the biomass of the MDR *S. aureus* in the biofilm. The expression of the genes encoding efflux pumps and biofilm (*nor* A, *nor* B, *cna*, *fnb* A, and *ica* genes) was significantly suppressed after exposure to ambroxol at the concentration of 1/4 MIC. We determined the effect of ambroxol on the burn infection to confirm its capability to be effective against the MDR *S. aureus* infection in vivo. Ambroxol enhanced wound healing and diminished the levels of TNF-α, which is an inflammatory marker [57–59]. Previous studies documented the inhibitory potential of ambroxol on signaling pathways of inflammation like nuclear factor kappa B (NF-κB) [33, 49, 60–62].

After all, the molecular docking studies revealed good affinities with five enzymes of *S. aureus*, with CrtM being the highest. Ambroxol showed good affinities, suggesting the possible mechanisms for its activity on *S. aureus*. Dehydrosqualene synthase or CrtM of *S. aureus* contributes to synthesizing golden carotenoid pigment staphyloxanthin. Such pigment has an antioxidant character that enables the bacteria to survive inside the host cell [63], which could be ambroxol’s antibacterial action mechanism.

## Conclusion

From the previous data, we can conclude that ambroxol inhibited the bacterial growth at 0.75 to 1.5 mg/mL and revealed an inhibitory action on the biofilm production of the MDR *S. aureus* isolates by crystal violet assay, SEM, and qRT-PCR. In an animal model, ambroxol confirmed its effectiveness as an antibacterial drug in the infected burn. Also, molecular docking studies have revealed the antibacterial consequence of ambroxol on *S. aureus*. Consequently, ambroxol should be investigated in the future in clinical reports as an antibacterial agent.

## Electronic supplementary material

Below is the link to the electronic supplementary material.

Supplementary Material 1

## Data Availability

All data generated or analyzed during this study are included in this published article and its supplementary information files.
