# Supplementary material for: Unveiling the antibacterial action of ambroxol against Staphylococcus aureus bacteria: in vitro, in vivo, and in silico investigation
Source: BMC Microbiol. 2024 Nov 29;24:507. doi: 10.1186/s12866-024-03666-x (PMC11606196; doi:10.1186/s12866-024-03666-x)
Supplement: Supplementary file 1 — Supplementary Material 1 [file 12866_2024_3666_MOESM1_ESM.docx]

*2.5. Biofilm inhibition assay*

The biofilm formation assay was applied according to Stepanovic et al. [[1](#_ENREF_1)] with some modifications (supplementary file). A tryptone soy broth (TSB) suspension was prepared from an over-night bacterial culture and it was adjusted to 10^6^ CFU/mL in freshly prepared TSB. Then, 200 µL of the bacterial suspension was added to the microtitration plates^,^ wells in the presence and absence of sub-MIC (0.5 MIC) of ambroxol and incubated at 37 °C for 48 h [[2](#_ENREF_2)]. The TSB was gently removed and the wells were washed to remove any planktonic cells and subsequently left for air drying. Add 200 µL of 99% methanol for 20 min then the formed biofilm was stained with 200 µL of 1% crystal violet (CV) solution for 15 min. After washing of the plate, 33% glacial acetic acid was utilized as a solvent for CV. Using microtitration plate reader (Sunrise, Männedorf, Switzerland) to measure the absorbance of the solubilized dye was measured at 570 nm.

*2.7. Gene expression measurement using qRT‑PCR*

The effect of ambroxol was studied on the expression levels of the biofilm and efflux genes (*cna*, *fnb*A, *ica*, *nor*A, *nor*B) by qRT-PCR (supplementary file). Primers used in this study are listed in Table S1 [[3](#_ENREF_3), [4](#_ENREF_4)]. After growing the isolates in TSB in the presence and absence of sub-MICs of ambroxol, they were incubated overnight at 37 °C. After the incubation period, cells were harvested by centrifugation and immediately stored at -80 °C. The total RNA from *S. aureus* isolates was extracted and purified using TRIzol^®^ reagent (Life Technologies, USA) following the manufacturer protocol. Reverse transcription was employed using QuantiTect Reverse Transcription kit (Qiagen, Germany). After that, the formed cDNA was amplified using Maximas SYBR Green/Fluorescein qPCR master mix (Thermo Fisher Scientific, USA).

The average threshold cycle (CT) values were normalized to the housekeeping gene (16s rRNA). The relative gene expression of the treated isolates was compared to that in the untreated ones according to the 2^−∆∆Ct^ method [[5](#_ENREF_5)].

**Table S1.** Sequences of the utilized primers

| **Gene** | **Forward Primer (5′ to 3′)** | **Reverse Primer (5′ to 3′)** |
| --- | --- | --- |
| *norA* | GACATTTCACCAAGCCATCAA | TGCCATAAATCCACCAATCC |
| *norB* | AGCCCCTTGTCTATCTTTCC | GCAGGTGGTCTTGCTGATAA |
| *ica*A (intercellular adhesion gene) | GAGGTAAAGCCAACGCACTC | CCTGTAACCGCACCAAGTTT |
| *fnb*A (fibronectin-binding protein A) | AAATTGGGAGCAGCATCAGT | GCAGCTGAATTCCCATTTTC |
| *cna* (collagen binding protein) | AATAGAGGCGCCACGACCGT | GTGCCTTCCCAAACCTTTTGAGC |
| 16S rRNA (housekeeping gene) | GGGACCCGCACAAGCGGTGG | GGGTTGCGCTCGTTGCGGGA |

**Table S2.** Impact of ambroxol on the biofilm forming ability of *S. aureus* isolates.

| Isolate code | Biofilm formation ability* | | Isolate code | Biofilm formation ability* | |
| --- | --- | --- | --- | --- | --- |
|  | Before treatment | After treatment |  | Before treatment | After treatment |
| S2 | MBF | WBF | S37 | MBF | NBF |
| S5 | MBF | NBF | S39 | MBF | WBF |
| S6 | MBF | WBF | S50 | MBF | NBF |
| S10 | MBF | NBF | S54 | MBF | WBF |
| S11 | MBF | NBF | S58 | MBF | NBF |
| S12 | MBF | NBF | S59 | MBF | WBF |
| S16 | MBF | WBF | S60 | MBF | NBF |
| S18 | MBF | NBF | S62 | MBF | WBF |
| S20 | MBF | NBF | S63 | MBF | NBF |
| S21 | MBF | WBF | S65 | MBF | NBF |
| S22 | MBF | WBF | S66 | SBF | WBF |
| S25 | MBF | NBF | S69 | MBF | WBF |
| S26 | MBF | WBF | S70 | MBF | WBF |
| S27 | MBF | NBF | S75 | MBF | WBF |
| S28 | MBF | WBF | S77 | MBF | NBF |
| S34 | MBF | NBF | S78 | SBF | NBF |
| S35 | SBF | NBF | S80 | SBF | NBF |
| S36 | MBF | NBF |  |  |  |

* SBF means strong biofilm forming, MBF means moderate biofilm forming, WBF means weak biofilm forming, and NBF means non-biofilm forming.


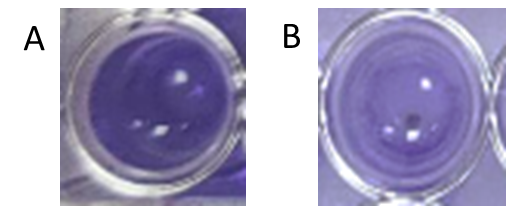


**Figure S1.** The effect of ambroxol on the biofilm forming ability of a representative *S. aureus* isolate: A) before treatment and B) after treatment.

**
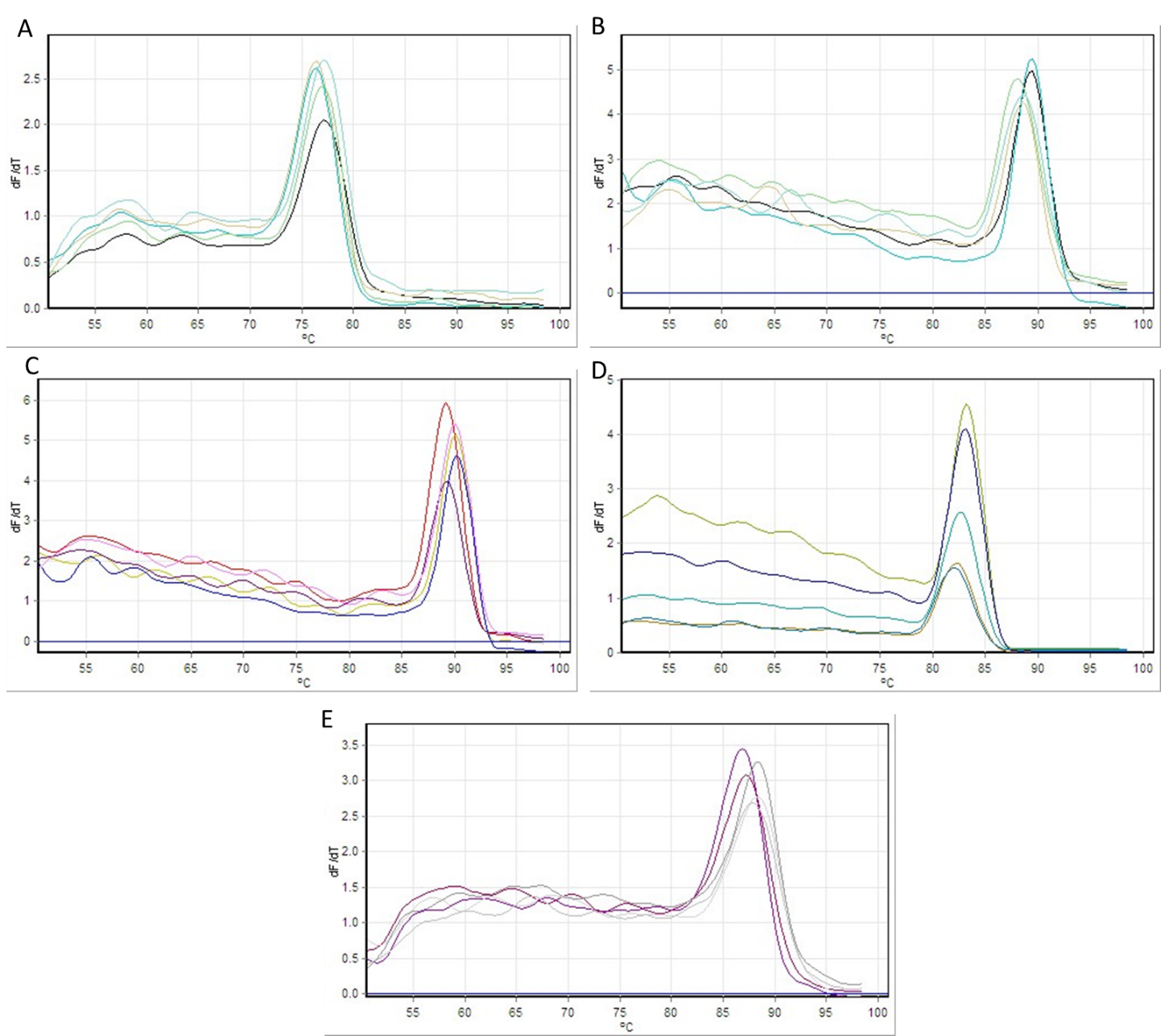
**

**Figure S2.** Melting curves for qRT-PCR A) *cna*, B) *fnb*A, C) *ica*, D) *nor*A, and E) *nor*B genes.

**Table S3.** The histological scoring of the experimental groups.

| **Groups** | **Mean count** | | |
| --- | --- | --- | --- |
|  | **Polymorphonuclear neutrophils** | **Granulation tissue (fibroblast, myofibroblast, neovascularization)** | **Fibrosis (collagen fibers)** |
| Normal control | 0 ± 1 | 0 ± 1 | 0.5 ± 1 |
| Positive control | 3 ± 0 | 3 ± 0 | 3 ± 1 |
| Standard drug-treated group | 1 ± 2 | 1 ± 1 | 1 ± 2 |
| Ambroxol-treated group | 1.5 ± 1 | 1.5 ± 2 | 2 ± 1 |

1. Stepanović, S.; Vuković, D.; Hola, V.; Bonaventura, G. D.; Djukić, S.; Ćirković, I.; Ruzicka, F., Quantification of biofilm in microtiter plates: overview of testing conditions and practical recommendations for assessment of biofilm production by staphylococci. *Apmis* **2007,** 115, (8), 891-899.

2. Abbas, H. A.; Atallah, H.; El-Sayed, M. A.; El-Ganiny, A. M., Diclofenac mitigates virulence of multidrug-resistant Staphylococcus aureus. *Archives of microbiology* **2020,** 202, 2751-2760.

3. Nejabatdoust, A.; Zamani, H.; Salehzadeh, A., Functionalization of ZnO nanoparticles by glutamic acid and conjugation with thiosemicarbazide alters expression of efflux pump genes in multiple drug-resistant Staphylococcus aureus strains. *Microbial Drug Resistance* **2019,** 25, (7), 966-974.

4. Mastoor, S.; Nazim, F.; Rizwan-ul-Hasan, S.; Ahmed, K.; Khan, S.; Ali, S. N.; Abidi, S. H., Analysis of the antimicrobial and anti-biofilm activity of natural compounds and their analogues against Staphylococcus aureus isolates. *Molecules* **2022,** 27, (20), 6874.

5. Livak, K. J.; Schmittgen, T. D., Analysis of relative gene expression data using real-time quantitative PCR and the 2− ΔΔCT method. *methods* **2001,** 25, (4), 402-408.
